# New Hosts of *Simplicimonas similis* and *Trichomitus batrachorum* Identified by 18S Ribosomal RNA Gene Sequences

**DOI:** 10.1155/2013/831947

**Published:** 2013-07-10

**Authors:** Kris Genelyn B. Dimasuay, Orlie John Y. Lavilla, Windell L. Rivera

**Affiliations:** ^1^Institute of Biology, College of Science, University of the Philippines, Diliman, 1101 Quezon City, Philippines; ^2^Molecular Protozoology Laboratory, Natural Sciences Research Institute, University of the Philippines, Diliman, 1101 Quezon City, Philippines

## Abstract

Trichomonads are obligate anaerobes generally found in the digestive and genitourinary tract of domestic animals. In this study, four trichomonad isolates were obtained from carabao, dog, and pig hosts using rectal swab. Genomic DNA was extracted using Chelex method and the 18S rRNA gene was successfully amplified through novel sets of primers and undergone DNA sequencing. Aligned isolate sequences together with retrieved 18S rRNA gene sequences of known trichomonads were utilized to generate phylogenetic trees using maximum likelihood and neighbor-joining analyses. Two isolates from carabao were identified as *Simplicimonas similis* while each isolate from dog and pig was identified as *Pentatrichomonas hominis* and *Trichomitus batrachorum*, respectively. This is the first report of *S. similis* in carabao and the identification of *T. batrachorum* in pig using 18S rRNA gene sequence analysis. The generated phylogenetic tree yielded three distinct groups mostly with relatively moderate to high bootstrap support and in agreement with the most recent classification. Pathogenic potential of the trichomonads in these hosts still needs further investigation.

## 1. Introduction

Protozoa are prevalent microorganisms found in almost all habitats worldwide. In the recent years, the classification of protozoa has been continuously undergoing successive improvements. The initial classification of protozoa was based on morphological and cytological observations using light and electron microscopy. However, with the recent advancements in molecular biology, DNA sequences, discrete features of gene organization, and biochemical properties have been used to provide phylogenetic classification of protozoa.

One of the well-known protozoan phyla is Parabasalia. The Parabasalia is composed of flagellated anaerobic protists defined by the presence of hydrogenosomes, flagellar apparatus, parabasal body, and parabasal filament. Species under parabasalids were grouped primarily according to the morphology and ultrastructural characteristics of their cytoskeletons. Recently, this highly encompassing phylum is divided into six classes based on both morphological and molecular phylogenetic analyses: Trichomonadea, Tritrichomonadea, Hypotrichomonadea, Cristamonadea, Spirotrichonymphea, and Trichonymphea [1]. Species of medical and veterinary importance are generally found in the classes Trichomonadea, Tritrichomonadea, and Hypotrichomonadea [2].

Trichomonads are obligate protozoan symbionts found in the digestive and genitourinary tract of animals. These organisms are believed to be part of the most primitive group of eukaryotic organisms [3, 4]. Moreover, they represent a well-defined monophyletic group adapted to live in micro- to anaerobic environments. Both commensal and parasitic trichomonads were found to exist [5]. Although some trichomonads are harmless, several of them were known for their pathogenicity most especially to humans and common domestic animals such as birds, bovines, canines, cats and swine. Some notable species of trichomonads include *Tritrichomonas foetus*, *Trichomonas gallinae,* and *Trichomonas vaginalis*. 

Little information is known about the trichomonads from human and animal hosts in the Philippines. Most of the published studies focused mainly on *T. vaginalis* [6–9]. In addition, a study about trichomonads from different animal hosts was published very recently [2]. Thus, this study aimed to identify the four trichomonad isolates collected from carabao, dog, and pig and to clarify the phylogenetic relationships of trichomonads using 18S ribosomal RNA gene sequence analysis. Also, this study sought to contribute to the identification, transmission, and epidemiology of the trichomonads.

## 2. Materials and Methods

### 2.1. Collection of Samples

Rectal swab samples were collected from different locations in the Philippines: two carabao (*Bubalus bubalis*) hosts from Laguna, one dog (*Canis familiaris*) host from Quezon City, and one pig (*Sus scrofa domestica*) host from Batangas using sterile cotton swabs. Animals used in the study were healthy at the time of collection. Swabs were inoculated in a diphasic medium composed of NaCl, Na_2_HPO_4_, KH_2_PO_4_, and L-asparagine supplemented with 10% heat-inactivated horse serum together with streptomycin penicillin at 50 *μ*g/mL [10] and incubated at 37°C overnight. Presumptive trichomonad growth was observed through its distinct tumbling motility under a light microscope. The morphologically identified trichomonad isolates were cultivated and maintained in culture at room temperature and subcultured at least once a week.

### 2.2. Extraction of DNA

Genomic DNA was extracted using Chelex method developed by Ong and Rivera [8]. Cells were collected by centrifugation in microfuge tubes at 9,604 ×g for two minutes. Pellets were washed with phosphate-buffered saline (pH 7.4) until visible contaminants were removed. Aggregated cells were resuspended in 5% Chelex 100 (Sigma-Aldrich, St. Louis, MO) and then mixed vigorously using a vortex. This was incubated at 56°C for 30 minutes and then to boiling water for eight minutes. Final centrifugation was done at 16,280 ×g for three minutes. The aqueous layer was then transferred into a new microfuge tube and stored at −20°C.

### 2.3. Polymerase Chain Reaction (PCR) and Sequencing

The PCR assay was done using Promega PCR Master Mix (Promega Corporation, Wisconsin, USA) and two sets of primers specific for the 18S ribosomal RNA gene of trichomonads [2]. The amplification cycle consisted of initial denaturation at 94°C for five minutes followed by 30 cycles of denaturation at 94°C for one minute, annealing at 56°C for one minute and polymerization at 72°C for two minutes. Final elongation step was carried out at 72°C for five minutes. Amplification products were separated by gel electrophoresis together with a positive control, *T. vaginalis*, on a 1.5% agarose gel at 100 V and detected by staining with ethidium bromide. It was then viewed under shortwave UV transillumination. Positive PCR products were sent to Macrogen Inc., (Seoul, South Korea) for purification and sequencing.

### 2.4. Phylogenetic Analysis

The DNA sequence traces of the trichomonad isolates were prepared, processed, and assembled using Staden Package [11]. Each of the isolates produced two preprocessed chromatograms since two sets of primers were used. Once disagreements like gaps and ambiguous sequences were already resolved, a consensus sequence was produced. Using the online BLASTN search function of NCBI GenBank, the exact match or closest similarity of the gene sequences of the trichomonad isolates were carried out against the database entries in order to determine and establish their identities. The phylogenetic position of the isolates relative to known trichomonads was determined by constructing phylogenetic trees rooted using *Trichonympha agilis *as the outgroup. *T. agilis* was used in view of its close relationship with trichomonads in terms of morphology and previous phylogenetic studies [12, 13]. Alignment of the isolate sequences and the retrieved sequences from the NCBI GenBank database was done using the CLUSTAL W algorithm function of BioEdit v. 7.0. The optimal model of DNA substitution was determined to be GTR (general time reversible) + Γ (gamma distribution of rates with 16 rate categories) using the jModeltest [14]. Saturation was also determined using Xia test in DAMBE [15]. Phylogenetic trees were then constructed using neighbor joining (NJ) and maximum likelihood (ML). PAUP∗ 4.0 was used to construct the NJ tree [16] while PhyML v. 2.4.4 [17] was used for the ML tree both with 1000 bootstrap replicates. Clusters were considered valid if the bootstrap support is greater than 50%. Phylogenetic trees were viewed using Tree Explorer v. 1.6.6 [18].

### 2.5. Nucleotide Sequence Accession Numbers

Sequences from this study were deposited and available in GenBank through accession numbers KC953858–KC953861 (Table 1).

## 3. Results

Four trichomonad isolates were obtained from carabao, dog, and pig hosts. The PCR assay using the two primer sets yielded positive products each with DNA fragments of estimated sizes of 900 bp and 800 bp (data not shown). 

NCBI BLASTN search function of the obtained DNA sequences showed very high similarity against homologous sequences of reference trichomonads (Table 1). The two carabao isolates (PCC3007 and PCC6005) were 94% and 95% similar with *Simplicimonas similis* GQ254637 isolated from gecko. Meanwhile, the dog isolate (D34) was identical with *Pentatrichomonas hominis* DQ412643 with 99% similarity. Lastly, the pig isolate (B266) showed 97% similarity with *Trichomitus batrachorum *AF124610. 

**Figure 1:**
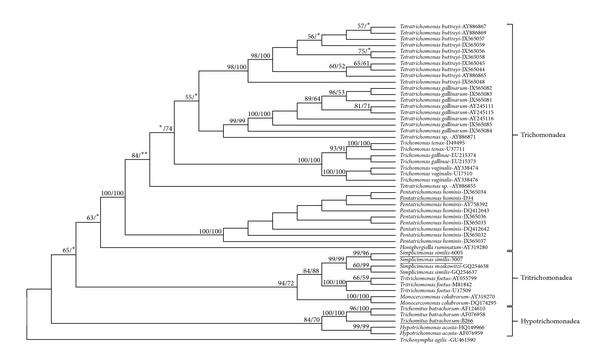
Phylogenetic tree of trichomonad isolates and reference gene sequences using maximum likelihood analysis. The tree was rooted on *Trichonympha agilis*. The values on the nodes are bootstrap support from maximum likelihood and neighbor-joining analyses, respectively. Bootstrap support lower than 50% is not shown. ∗: weak support; ∗∗: branch not observed in NJ tree.

The 18S rRNA gene sequences of the four isolates together with 48 reference sequences retrieved from NCBI GenBank including *T. agilis* that served as an outgroup were aligned to generate maximum likelihood and neighbor-joining phylogenetic trees (Figure 1). The phylogenetic tree identified three groups (Trichomonadea, Tritrichomonadea, and Hypotrichomonadea) supported by moderate to high bootstrap values. The first group consisted of reference sequences from class Trichomonadea along with the dog isolate (D34) that clustered highly with *P. hominis*. The second group consisted of species from Tritrichomonadea including the carabao isolates (PCC6005 and PCC3007) that grouped with high bootstrap support to *Simplicimonas *sp. Moreover, the third group formed the Hypotrichomonadea with the pig isolate (B266) that clustered with *T. batrachorum*. The consensus tree was rooted to *T. agilis *GU461590.

## 4. Discussion

 In this study, trichomonads were successfully isolated from rectal swabs of carabao, dog, and pig. The results of the PCR demonstrated that the two novel primer sets, T18SF and T18SRi and T18SFi, and T18SR can be used for the specific and sensitive detection of trichomonads [2]. A phylogenetic tree was constructed using maximum likelihood and neighbor-joining analyses. Moderate to high bootstrap values were readily observed in the established groups indicating high support and enhanced reliability of the generated tree. Moreover, the constructed phylogenetic tree coincided with the results of the NCBI BLASTN queries of the isolates.

The first group formed the class Trichomonadea. According to Cepicka et al. [1], the class splits into two orders, Trichomonadida and Honigbergiellida. This was observed in the consensus tree with species from the order Trichomonadida forming a highly supported clade with both 100% ML and NJ support while *Honigbergiella ruminatum*, a representative from Honigbergiellida, was placed independently between the clades and thus showing 64% ML bootstrap support only. Similarly, the family Trichomonadidae under the order Trichomonadida is divided into two robust clades, the *Trichomonas*-group and the *Pentatrichomonas*-group. As shown in the consensus tree, the dog isolate (D34) grouped with high bootstrap support to the *Pentatrichomas*-group. The *Pentatrichomonas*-group also formed a very distinct clade from the *Trichomonas*-group and was placed as the most basal taxon which in previous studies by Kleina et al. [19] and Cepicka et al. [1] were also clearly demonstrated.

*P. hominis *is considered a commensal protozoan in the large intestine of mammalian hosts, such as cats [20], dogs [21, 22], and nonhuman primates [22]. However, recent studies reported that *P. hominis *is an emerging threat to dogs causing the most common trichomoniasis infection [23]. Although its pathogenic effect remains unknown, it is believed that their opportunistic overgrowth can result in diarrheic infection. Hence, this suggests that the dog host used in this study could be asymptomatic from *P. hominis *infection. 

Meanwhile, the second group or class Tritrichomonadea formed a strongly supported clade showing bootstrap values of 94% and 72% for ML and NJ, respectively. Four families belonged to class Tritrichomonadea [1]: Tritrichomonadidae, Simplicimonadidae, Monocercomonadidae, and Dientamoebidae. In the phylogenetic tree, distinct clades of the three families were observed. Under the family Simplicimonadidae, the carabao isolates (PCC6005 and PCC3007) clustered in this clade with high support values in conjunction with the BLAST result. Cepicka et al. [1] first described *S. similis *from a gecko (*Uroplatus lineatus*). Another study showed the presence of *Simplicimonas *sp.-like organism in backyard chickens [24]. To our knowledge, this is the first report of *S. similis *in carabao. This could suggest that carabao is also a type host of *S. similis*. In addition, this could possibly mean the adaptation of *S. similis *to a new host. Thus, further investigation is warranted to detect, identify, and characterize this organism. Its pathogenicity and infectivity are also important to examine whether this protozoon can cause a disease or infection especially in carabao. Carabao industry is a major contributor to the total agricultural economy of the Philippines. 

Lastly, the third group formed a supported class Hypotrichomonadea (84% ML and 70% NJ). Hypotrichomonadea contains two genera, *Trichomitus* and *Hypotrichomonas*, which is highly supported by both ITS and SSU rRNA analysis [1]. In the study of Dimasuay and Rivera [2], both *T. batrachorum *and *H. acosta *were detected and identified in reptile hosts like iguanas, boa constrictors, lizards, and pythons. However, in the present study, *T. batrachorum *was identified in a pig (B266) with 97% sequence similarity in BLAST and with high bootstrap support values (100% ML, 100% NJ) in the consensus tree. A study by Mostegl et al. [25] also reported the presence of *T. batrachorum *in formalin-fixed and paraffin-embedded tissue sections in pigs. This appears to be the first report of *T. batrachorum *in live pigs using direct rectal swab. Thus, this raises a question as to whether *T. batrachorum *adapt to a new host, from reptile to mammal. Additional samples are required to confirm this finding.

## 5. Conclusion

The occurrence of *S. similis* in carabao and *T. batrachorum *in pig is an evidence that trichomonads have the capability to adapt to new hosts. Therefore, zoonotic transmission is possible to happen especially for these domestic animals wherein humans play a major role for their survival. Although pathogenesis is yet to be explored for *S. similis *and *T. batrachorum*, this suggests the possibility of being a new pathogen or causative agent of infection for these animals. The animals used in this study were free from any disease implying that they were either commensal or asymptomatic. It is therefore recommended that a more extensive sampling be done to fully confirm the presence of the trichomonads in these new hosts and to investigate further their pathogenic potential.

Furthermore, the 18S rRNA gene analysis done in this study could therefore be used to generate a reliable and supported identity of the isolates. The constructed phylogenetic tree is in accordance with the new classification of Cepicka et al. [1]. Still, the assignment of the isolates to their corresponding taxon needs further verification by supplemental results from methods other than the 18S rRNA gene analysis.

## Figures and Tables

**Table 1:** Trichomonad isolates and their respective homologous sequences from NCBI BLASTN query search and percent homology to DNA samples.

Sample code	Domestic animal host	GenBank accession number	BLAST result			
			Identity	Strain host	Length	Sequence similarity (%)
PCC3007	Carabao	KC953858	*Simplicimonas similis* (GQ254637)	Gecko	1492 bp	94 (1358/1448)
PCC6005	Carabao	KC953859	*Simplicimonas similis* (GQ254637)	Gecko	1492 bp	95 (1410/1480)
D34	Dog	KC953860	*Pentatrichomonas hominis* (DQ412643)	Cattle	1513 bp	99 (1492/1495)
B266	Pig	KC953861	*Trichomitus batrachorum* (AF124610)	NS	1502 bp	97 (1438/1481)

NS: not specified.
